# Functional Interplay between Arginyl-tRNA Synthetases and Arginyltransferase

**DOI:** 10.3390/ijms231710160

**Published:** 2022-09-05

**Authors:** Irem Avcilar-Kucukgoze, Brittany MacTaggart, Anna Kashina

**Affiliations:** School of Veterinary Medicine, University of Pennsylvania, Philadelphia, PA 19104, USA

**Keywords:** arginylation, ATE1, tRNA, RARS, Arg-tRNA synthetase

## Abstract

Protein arginylation, mediated by arginyltransferase ATE1, is a post-translational modification of emerging biological importance that consists of transfer of the amino acid Arg to protein and peptide substrates. ATE1 utilizes charged tRNA^Arg^ as the donor of the arginyl group, which depends on the activity of Arg-tRNA synthetases (RARS) and is also utilized in translation. The mechanisms that regulate the functional balance among ATE1, RARS and translation are unknown. Here, we addressed the question of how these two enzymes can partition Arg-tRNA^Arg^ to functionally distinct pathways using an intracellular arginylation sensor in cell lines with overexpression or deletion of ATE1 and RARS isoforms. We found that arginylation levels depend on the physiological state of the cells but are not directly affected by translation activity or the availability of RARS isoforms. However, displacement of RARS from the multi-synthetase complex leads to an increase in intracellular arginylation independently of RARS enzymatic activity. This effect is accompanied by ATE1′s redistribution into the cytosol. Our results provide the first comprehensive analysis of the interdependence among translation, arginyl-tRNA synthesis and arginylation.

## 1. Introduction

Protein arginylation, mediated by arginyltransferase ATE1, is a biological regulatory mechanism that involves the addition of the amino acid Arg to proteins and peptides [1]. Over 100 in vivo arginylation targets have been identified [2,3,4,5,6,7], implicating ATE1 in the regulation of diverse cellular and organismal processes, including cell migration [8], nucleotide biosynthesis [9], neurodegeneration [2] and cancer [10]. ATE1 is encoded by a single gene in animals and fungi, and by two genes in plants. In higher vertebrates, the *Ate1* gene generates four alternatively spliced isoforms [11]. It is unclear how these four highly similar enzymes can perform a multitude of diverse in vivo functions, and what intracellular factors contribute to this regulation. To date, very few ATE1 functional partners have been identified, and very little is known about the intracellular mechanisms that balance arginylation with other pathways that utilize the same molecules as substrates, such as protein synthesis.

ATE1 requires Arg conjugated to tRNA^Arg^ as the donor of the arginyl group, and thus, arginylation directly depends on the activity of arginyl-tRNA synthetases (RARS). In principle, this dependence places arginylation in direct competition with translation, which also depends on RARS, along with other aminoacyl-tRNA synthetases (AARSs), to provide aminoacyl-tRNA (aa-tRNA) for the growing polypeptide chains. Previous work from our lab showed that, in addition to full-length tRNA^Arg^, ATE1 can utilize Arg-conjugated tRNA^Arg^-derived fragments (tRF^Arg^), which are translation-incompetent and thus can potentially serve to shift the balance between translation and arginylation [12]. However, generation of Arg-tRF^Arg^ also requires RARS as the initial step, and thus RARS availability and activity can potentially be rate-limiting for arginylation.

In mammalian cells, RARS exists as two isoforms derived from one mature mRNA [13,14] via translation from two alternative start codons. Thus, these two RARS isoforms are identical downstream of the second start codon, but the “long” RARS includes an additional N-terminal stretch of sequence containing a leucine zipper (LZ) that scaffolds this RARS into the multi-tRNA synthetase complex (MSC, which contains IARS, LARS, MARS, QARS, RARS, KARS, DARS, EPRS and three scaffold proteins: AIMP-1, -2 and -3) [15]). This complex is largely dedicated to translation [16]. In contrast, the “short” RARS, which lacks this domain, is soluble and cytosolic. The MSC has been proposed to channel aa-tRNAs directly to ribosomes to support efficient translation [16]. The LZ of long RARS interacts with AIMP-1, which is required for the assembly of RARS in the MSC. This interaction also forms a platform to anchor QARS to the MSC [17]. Although AARSs are exclusively located in the cytoplasm of eukaryotic cells to provide protein synthesis, several studies revealed that MSC-bound AARSs have been also found in the nucleus [17,18]. It has been previously proposed, on the basis of this scaffolding, that the long RARS functions primarily in translation, while the short RARS could potentially be partially or exclusively dedicated to translation-independent functions, including arginylation [14]; however, this hypothesis has never been directly tested. It was recently found that displacement of the long RARS from the MSC by deletion of the leucine zipper domain responsible for this scaffolding does not affect global translation levels or tRNA^Arg^ aminoacylation [17], suggesting that the balance between translation and potential translation-independent RARS functions might be more complex.

Here, we used in vitro and in vivo assays to investigate how these two enzymes can partition Arg-tRNA^Arg^ to functionally distinct pathways, including arginylation, translation and the balance of long and short RARS isoforms. Our results demonstrated that intracellular arginylation activity does not directly depend on active translation or the levels of RARS enzymes. However, we found that displacement of the long RARS from the MSC increases intracellular arginylation. In the absence of the long RARS, ATE1 redistributes into the cytosolic fraction in cells, reminiscent of a similar redistribution of RARS [17]. Our results suggest that ATE1 is linked to non-canonical RARS functions in driving the redistribution of MSC into the cytosol, and they provide the first comprehensive analysis of the interdependence among translation, arginyl-tRNA^Arg^ synthesis and arginylation.

## 2. Results

### 2.1. Intracellular Measurements of Arginylation Using an Arginylation Sensor Plasmid

**Figure 1:**
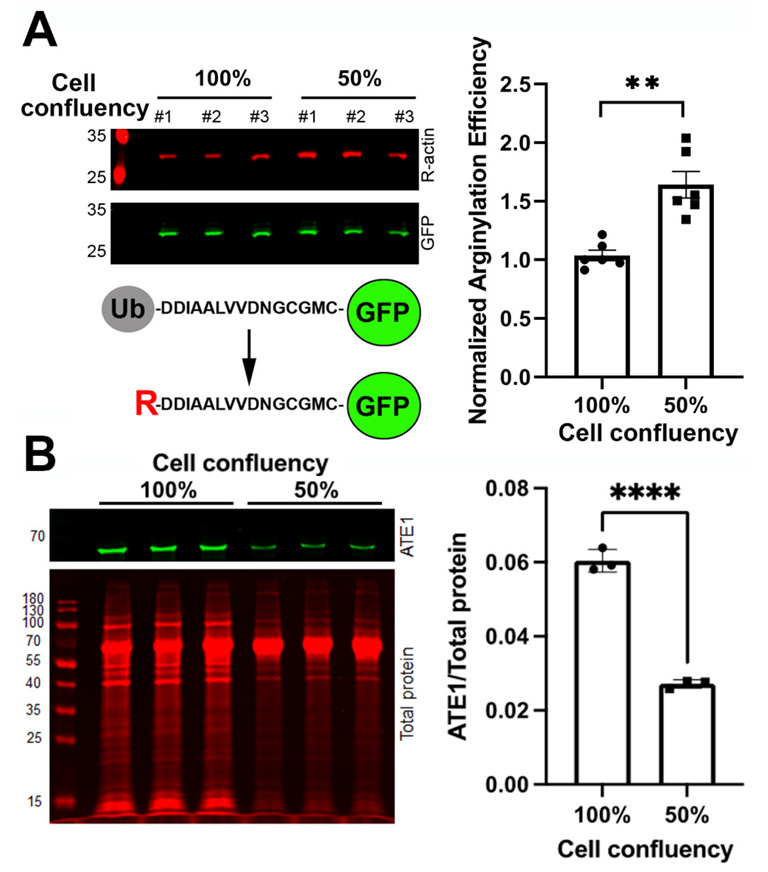
Intracellular arginylation activity depends on the physiological state of the cell. (**A**) Left, immunoblots (top) and schematic representation (bottom) of the arginylation sensor used in this study to detect intracellular arginylation. In this arginylation sensor, a 15-residue peptide based on the β-actin N-terminal sequence is expressed in cells as a fusion with an N-terminal ubiquitin (Ub) moiety and a C-terminal GFP. Arginylation occurs on the N-terminal D of the sequence following cotranslational Ub removal by deubquitinating enzymes and is detected with antibodies against the arginylated β-actin (R-actin). Right, quantification of arginylation activity as the ratio of R-actin to GFP signal in human embryonic kidney 293T cells grown to 100% or 50% confluency. Error bars represent SEM; n = 6 biological replicates. ** *p* < 0.01, Welch’s *t*-test. (**B**) Quantification of the ATE1 levels in 100% and 50% confluent cell cultures. Left, Western blot showing the ATE1 signal (green, top) and total protein stain (red, bottom) in three independently loaded 100% confluent (left three lanes) and 50% confluent (right three lanes) cultures of mouse embryonic fibroblasts. Right, quantification of the ATE1 levels in these cultures normalized to total protein levels. Error bars represent SD; n = 3 independent repeats; **** *p* < 0.001, Student’s *t*-test.

Early studies characterizing N-terminal arginylation as a modification that primarily targets the acidic N-terminal residues used an N-terminal ubiquitin (Ub) fusion technique. In this approach, an N-terminal Ub moiety was added to the N-terminus of the target protein and was then co-translationally removed by deubiquitinating enzymes to expose the next residue at the protein’s N-terminus [19]. This approach enabled the intracellular expression of proteins with any N-terminal residue. Prior studies found that Ub removal from such constructs is highly efficient in different cell types [20]. Furthermore, no N-terminal Ub moiety can be detected on β-actin variants expressed with the N-terminal Ub-Met or Ub-Asp sequence in confluent and semi-confluent cells [8,21]. Recently, this technique was used to develop an arginylation sensor, based on the intracellular expression of an actin-derived N-terminal arginylation target peptide fused to GFP [22]. Using these constructs in cells enables ratiometric imaging of arginylation levels using Arg- and GFP-specific antibodies ([22] and Figure 1A, left). While the similarity of the peptide target sequence with actin may potentially bias this arginylation sensing toward the actin-based pathway(s), such a sensor currently represents the only reliable tool that can measure intracellular arginylation and its changes in response to physiological conditions and cellular stimuli.

### 2.2. Intracellular Arginylation Depends on the Physiological State of the Cell but Does Not Compete with Active Translation

We previously found that arginylation of β-actin, a process linked to the migratory activity of cells, significantly increases in 50% confluent cell cultures, where cells are expected to undergo active division and migration [23]. To test whether changes in cell confluency are accompanied by general changes in arginylation activity, as detected by the sensor described above, we used this sensor to test arginylation levels in confluent and semi-confluent cell cultures. In these assays, arginylation was significantly higher in semi-confluent cells compared with cells grown to a confluent monolayer (Figure 1A). To test whether this change relates to an increased abundance of ATE1 in semi-confluent cultures, or a genuine increase in ATE1 activity, we compared the ATE1 levels in both cultures and found that, remarkably, ATE1 was around threefold more abundant in confluent versus semi-confluent cultures (Figure 1B). Thus, semi-confluent conditions lead to activation of arginylation nearly sixfold higher compared with confluent cultures. Although, due to the similarity between the arginylation sensor plasmid and the actin N-terminus, this increase could potentially be biased toward the actin-dependent arginylation pathway (which is believed to facilitate cell migration in semi-confluent cultures), these data overall strongly suggest that the physiological state of cells can affect their arginylation activity.

**Figure 2:**
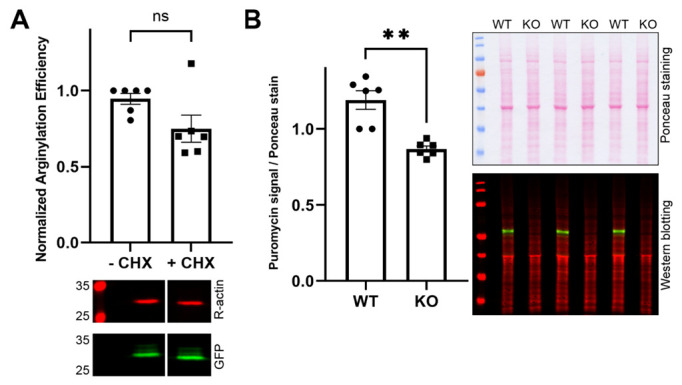
Intracellular arginylation depends on the physiological state of the cell but does not compete with active translation. (**A**) Chart (top) and representative immunoblots (bottom) of arginylation sensor quantification in human embryonic kidney 293T cells with and without the addition of cycloheximide (CHX) and chloramphenicol. Error bars represent SEM; n = 6. ns, not significant, Welch’s *t*-test. (**B**) Chart (left) and immunoblots (right) of wild-type (WT) and *Ate1* knockout (KO) mouse embryonic fibroblasts stained with Ponceau S for total protein load (top), and puromycin (red) and ATE1 antibodies (green) (bottom). Ratios of total puromycin signal from the entire lane to the Ponceau S signal in the same lane normalized to the control were plotted. Error bars represent SEM; n = 6 biological replicates. ** *p* < 0.01, Welch’s *t*-test.

One of the expected changes during cell transition to confluency is a decrease in metabolic activity [24] and translation, a process that utilizes tRNA^Arg^ and RARS activity, which are also required for arginylation by ATE1. Since confluent cells have lower arginylation activity, it is possible that this reduction may be a direct result of the reduction in active translation, which can conceivably be coupled, e.g., to reduced production of tRNA^Arg^. To test this and to find out if arginylation levels change upon translation inhibition, we compared the sensor arginylation levels in control cells and cells treated with the translation inhibitors cycloheximide and chloramphenicol. We reasoned that if arginylation and translation exist in direct competition, cycloheximide/chloramphenicol treatment should increase arginylation. If, however, arginylation depends on active translation as a donor of reactive compounds, such as Arg-tRNA^Arg^, we should see a decrease in arginylation after cycloheximide/chloramphenicol treatment. However, cycloheximide/chloramphenicol treatment did not change arginylation levels in cultures with similar confluency (Figure 2A). Thus, inhibition of translation does not appear to affect arginylation.

To confirm this, we also addressed a reciprocal possibility by testing whether the inhibition of arginylation affects translation activity. To do this, we measured the overall translation levels in *Ate1* knockout mouse embryonic fibroblasts, previously derived in our lab from *Ate1* knockout mice [9]. As a measure of translation activity, we used puromycin, which mimics the 3′ adenosine of a charged tRNA and is incorporated into the C-terminus of elongating nascent chains, thus labeling the entire body of newly synthesized proteins in the cell [25,26,27,28]. We reasoned that since *Ate1* knockout cells lack arginylation, if direct competition between translation and arginylation exists, these cells should exhibit an increase in translation activity in the absence of ATE1. However, in these assays, overall levels of puromycin staining normalized to Ponceau S staining were significantly lower in *Ate1* knockout cells compared with the wild-type (Figure 2B), suggesting that the lack of ATE1 in these cells impairs protein synthesis.

Thus, arginylation activity shows an overall dependence on the cells’ physiological state and facilitates translation, but there is no direct competition between translation and arginylation.

### 2.3. ATE1 Can Interact with the Long and Short RARS, but Overexpression of RARS Does Not Facilitate Arginylation

**Figure 3:**
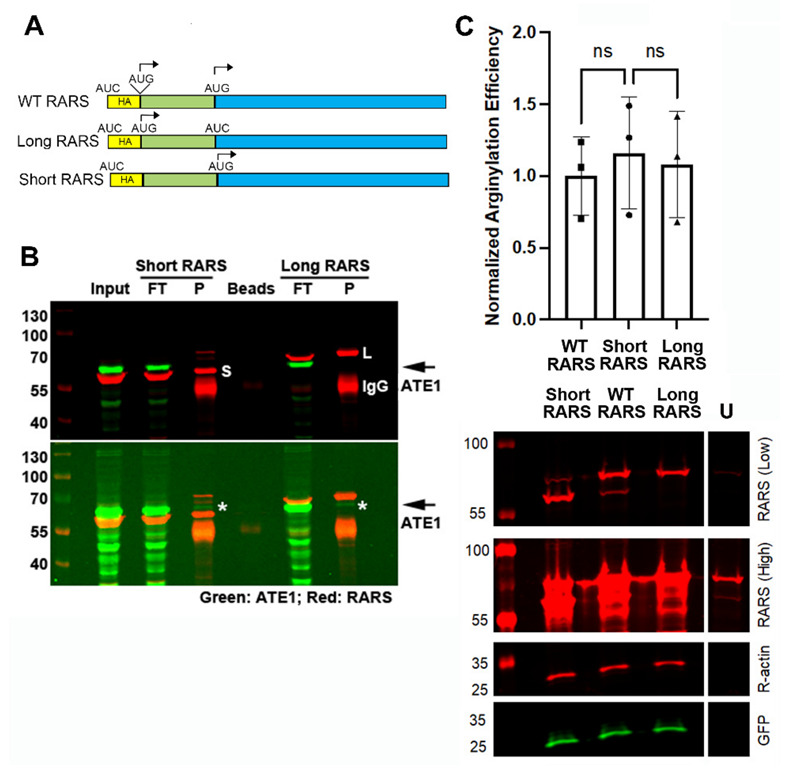
ATE1 can interact with the long and short RARS, but overexpression of RARS does not facilitate arginylation. (**A**) Schematic representation of the RARS constructs used for immunoprecipitation and overexpression. Start codons (AUG) were individually mutated to AUC to abolish the HA tag from the expression construct and ensure the start of translation either on both sites (WT) or on the upstream or downstream AUG individually (for the long and short RARS, respectively). (**B**) Representative immunoblots of the immunoprecipitation steps from HEK293T cells transfected with either the short or long RARS as indicated on the top and visualized with RARS and ATE1 antibodies. FT, flow-through; P, pulldown. The top and bottom panels show the same blot at different exposures to visualize the typical immunoprecipitation steps (top), as well as the minor levels of ATE1 in the precipitates (bottom, marked with asterisks and arrows on the right). The positions of the short and long RARS are indicated in the top panel as S and L, respectively. IgG, immunoglobulin heavy chain. The pulldowns were repeated at least 3 independent times with similar results. (**C**) Chart and representative immunoblots of arginylation sensor quantification in HEK293T cells transfected with different RARS isoforms as indicated. U, untransfected control. High and low exposure images of the same gel are shown to enable a comparison of the levels of endogenous RARS with the transfected RARS isoforms. Error bars represent SD; n = 3 biological replicates. ns, not significant, one-way ANOVA.

It has been previously proposed in early biochemical studies that ATE1 in vivo is complexed with RARS [29] and that it may functionally interact with the short RARS in the cytosol [14], even though direct interaction between ATE1 and RARS has never been demonstrated in any subsequent studies. To test whether ATE1 can directly interact with either of the RARS isoforms, we performed immunoprecipitation using RARS antibodies from cells overexpressing either the long or short RARS, generated by editing out either the first or the second translation initiation site in the RARS transcript (Figure 3A). We then tested these immunoprecipitation steps with antibodies against ATE1. In these assays, ATE1 was present mostly in the input and the flow-through (Figure 3B, top); however, a very minor amount of ATE1 could be detected in both the long RARS and short RARS immunoprecipitates (Figure 3B, bottom). This amount was not stoichiometric to RARS and pushed the overall detection levels revealed by Western blotting, suggesting that only a minor amount of ATE1 can potentially be involved in RARS interactions. Thus, the majority of both short and long RARS likely exists in the ATE1-free pool. Moreover, the minor ATE1 fraction found in the RARS precipitates did not exhibit any apparent bias toward the long or short RARS isoform.

To test whether the increased availability of either the short or long RARS can facilitate arginylation, we tested sensor arginylation levels in cells overexpressing the long RARS, the short RARS or the wild-type RARS transcript containing both alternative start codons that give rise to the two RARS isoforms. There was no difference in the arginylation levels between these cells (Figure 3C). Thus, overexpression of the different isoforms of RARS does not facilitate arginylation.

### 2.4. The ATE1–RARS Interaction Is tRNA-Independent

Both ATE1 and RARS are capable of direct interaction with tRNA^Arg^, and thus tRNA can potentially mediate the weak ATE1–RARS binding seen in our pulldowns. To test this possibility, we tested the interaction of purified ATE1 and RARS alone or with the addition of tRNA^Arg^ in the absence or presence of added cell extracts. Since both enzymes can bind tRNA^Arg^ and are expected to contain tRNA when purified from cells, we also performed similar pulldowns with the addition of RNase A to ensure that these pulldowns were RNA-free.

**Figure 4:**
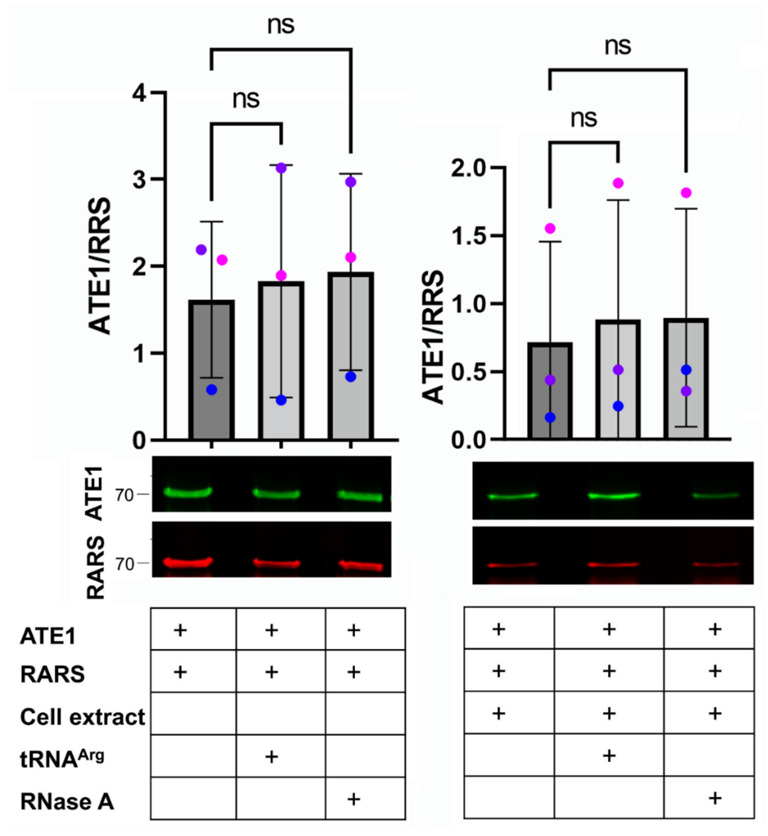
The ATE1–RARS interaction does not depend on tRNA. Quantification (top) and representative immunoblots (bottom) of purified ATE1 and RARS pulldowns in the presence and absence of tRNA, RNase A and cell extract, as indicated in the table on the bottom under each lane and bar (+ represents addition of each component in each line). Error bars represent SD; n = 3 independent pulldowns. ns, not significant, one-way ANOVA.

In these in vitro assays, RARS and ATE1 were mixed in equimolar amounts to ensure no bias. Under these conditions, RARS and ATE1 exhibited a clearly detectable interaction; however, this interaction was neither facilitated nor reduced by the addition of tRNA or RNase A (Figure 4, left). The addition of cell extract did not change this balance (Figure 4, right). Thus, tRNA does not affect ATE1–RARS binding.

### 2.5. Displacement of the Long RARS from the MSC Increases Intracellular Arginylation

**Figure 5:**
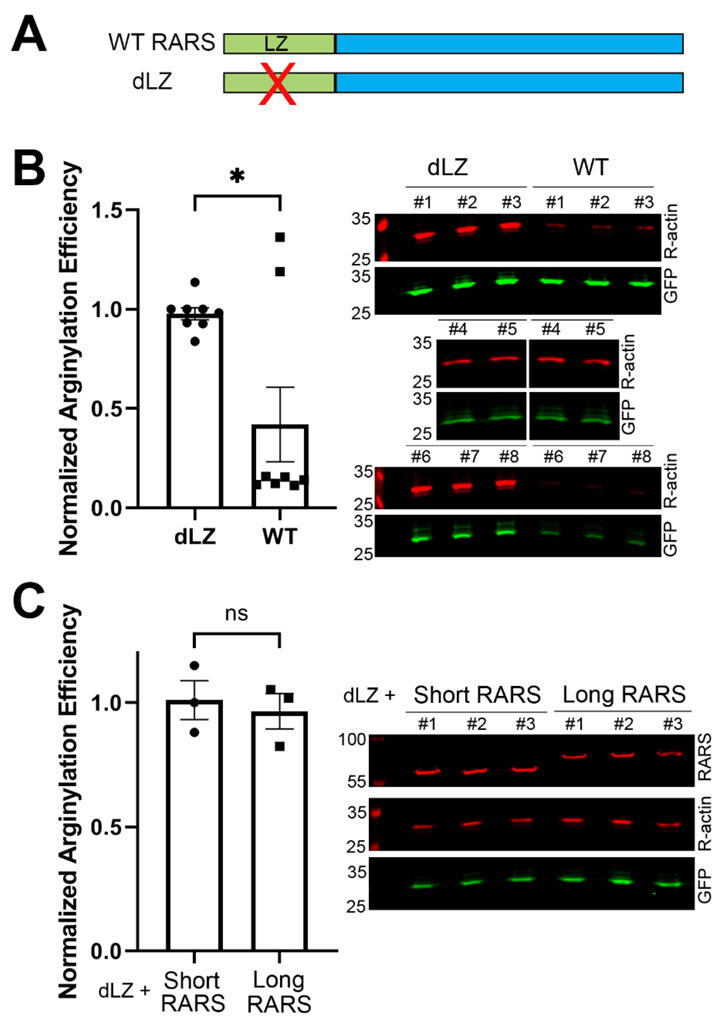
Displacement of the long RARS from the multi-synthetase complex increases intracellular arginylation. (**A**) Diagram representing the deletion of the leucine zipper (LZ) in dLZ cells to convert the entire RARS into the cytosolic soluble pool. (**B**) Chart and representative immunoblots of arginylation sensor quantification in HEK293T cells produced by knocking out the leucine zipper domain in the long RARS to displace it from the multi-synthetase complex (dLZ) compared with the parental cell line (WT). Cells of similar confluency were chosen as pairs for quantification. Error bars represent the SEM; n = 8. * *p* < 0.05, Welch’s *t*-test. (**C**) Chart and representative immunoblots of arginylation sensor quantification in dLZ cells transfected with the long or short RARS. Error bars represent the SEM; n = 3 biological replicates. ns, not significant, Welch’s *t*-test.

To test whether the redistribution of RARS between the soluble cytosolic (free) fraction and the fraction incorporated into the MSC affected arginylation, we used a previously produced cell line, in which the leucine zipper in the long RARS has been deleted, thus converting the entire RARS pool into the free (short) form [17]. In these cells, named dLZ for delta leucine zipper, RARS levels were similar to the control, but all of the RARS was represented by the short isoform that is unable to scaffold into the MSC complex (Figure 5A). Surprisingly, previous studies showed that this change does not affect translation [17]. However, the entire MSC is restricted from localizing into the nucleus, leading to the original conclusion by the authors of this study that RARS localization via the leucine zipper is required for the regulation of the alternative translation-independent nuclear functions of this enzyme [17].

To test whether the dLZ cells, which lack the long MSC-localized RARS, exhibited any changes in arginylation, we compared arginylation levels in these dLZ cells and the parental wild-type cells of similar confluency using the arginylation sensor. This comparison revealed a significant increase in arginylation in dLZ cells compared with the control cells (Figure 5B). This result suggests that the conversion of intracellular RARS from MSC-bound to the free pool directly or indirectly facilitates arginylation. At the same time, transfection of dLZ cells with either the short or long RARS did not affect arginylation (Figure 5C), consistent with our observation in control cells (Figure 3C). Together, these results suggest that while displacement of RARS from the MSC into the free pool facilitates arginylation, this effect is not related to RARS availability overall.

### 2.6. Displacement of RARS from the MSC Increases the Cytosolic Fraction of ATE1

**Figure 6:**
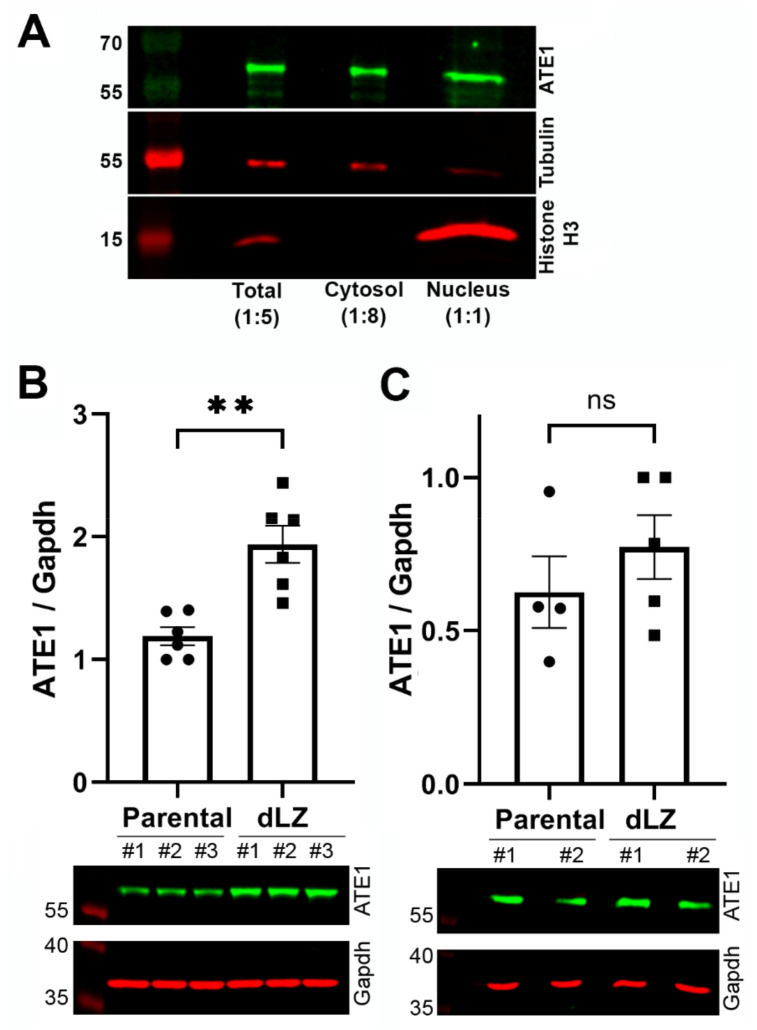
Displacement of the long RARS from the multi-synthetase complex induces ATE1 redistribution into the cytosol. (**A**) Sample fractionation showing the relative levels of ATE1 in the total extract (total), the cytosol and the nuclear fraction, probed with tubulin (a largely cytosolic marker) and histone H3 (a nuclear marker) to confirm the fractionation efficiency. (**B**) Chart and representative immunoblots of ATE1 levels in the cytosol of wild-type (parental) and dLZ cells. (**C**) Chart and representative immunoblots of total ATE1 levels in wild-type and dLZ cells. Error bars represent the SEM; n = 3 biological replicates. ** *p* < 0.01, Welch’s *t*-test.

In dLZ cells, the only effect on RARS found in the previous study was independent of its enzymatic activity and its role in translation, but linked to its non-canonical role in facilitating the localization of MSC to the nucleus [17]. Two of the four ATE1 isoforms have been previously shown to exhibit partial, transient nuclear localization [11,30,31]. Making a parallel with the RARS, it is conceivable that deletion of the leucine zipper and the ensuing displacement of AARSs from the nucleus may also affect the nuclear:cytosolic balance of ATE1. To test this hypothesis, we compared the levels of ATE1 in the cytosol and nucleus (Figure 6A), as well as in the cytosolic fraction in dLZ cells and control cells (Figure 6B). Overall, ATE1 in the nucleus constituted only a minor fraction of the total ATE1, around eightfold less abundant than in the cytosol (Figure 6A) and very difficult to detect in routine experiments without adjusting the protein load. At the same time, the ATE1 cytosolic fraction was significantly increased in dLZ cells (Figure 6B), while the overall ATE1 level in these cells was not changed (Figure 6C). Thus, displacement of RARS from the MSC facilitates ATE1 redistribution into the cytosol. We speculate that this change likely leads to the increased arginylation in dLZ cells (Figure 6A), since the arginylation sensor we are using is expected to be cytosolic.

## 3. Discussion

**Figure 7:**
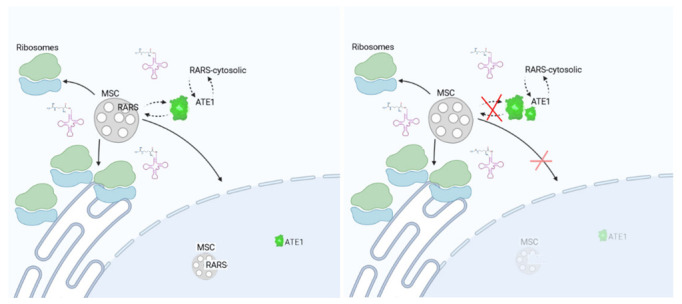
Interplay between ATE1 and RARS. In wild-type cells (**left**), ATE1 exists in a balance with free and MSC-bound RARS. Knockout of the leucine zipper (**right**) that scaffolds the long RARS to the MSC perturbs this balance by reducing the nuclear MSC and ATE1 localization, resulting in an increase in cytoplasmic ATE1 activity. Cytosolic and nuclear environments are denoted with white and blue backgrounds, respectively.

This work represents the first study of the mechanisms by which ATE1 and RARS partition Arg-tRNA^Arg^ to functionally distinct pathways to facilitate the interplay among arginylation, translation activity and RARS enzymes. We found that arginylation does not directly compete with the translation machinery and is not directly dependent on intracellular RARS levels but is functionally linked to RARS in a translation-independent manner, potentially facilitated by a direct or indirect ATE1–RARS interaction (Figure 7). Identification of the components of this interaction constitutes an exciting direction for future studies.

Our data revealed that intracellular arginylation activity depends on the physiological state of the cell and is higher in semi-confluent cells active in the cell cycle compared with resting cells in the dense monolayer. This finding is in agreement with our previously published data demonstrating a similar increase in β-actin arginylation in 50% confluent cell cultures compared with cells grown to 100% confluency [23]. While the specific factors driving this difference remain to be identified, this finding strongly suggests the existence of upstream mechanisms that regulate arginylation at the global cellular level. Investigating the contribution of individual pathways to this regulation will shed light on the overall role of arginylation.

ATE1 has been previously proposed to act in direct competition with the translation machinery, since it utilizes the same molecule, Arg-conjugated tRNA^Arg^, as the ribosome [12]. Our data show that the inhibition of translation does not affect intracellular arginylation levels. Notably, cycloheximide/chloramphenicol treatment has been used in multiple prior studies to suppress the incorporation of Arg into proteins during translation and to identify specific proteins that incorporate Arg through post-translational arginylation (see, e.g., [7,32]); however, these studies never addressed whether cycloheximide treatment changes arginylation levels in cells. Our current result strongly suggests that there is no direct competition between translation and arginylation. Since over 90% of the tRNA^Arg^ molecules in cells are charged with Arg [33], it is possible that they are sufficiently abundant for both pathways. It is also possible that ATE1 depends largely on translation-incompetent Arg-tRF^Arg^, previously demonstrated to be an efficient donor of the Arg group for arginylation [12].

We find that depletion of ATE1 leads to overall lower translation activity in cells. Given our data suggesting that ATE1 does not directly compete with protein synthesis, it seems likely that this effect is due to other mechanisms that may regulate translation through arginylation. It is possible, for instance, that the components of the translation machinery are directly arginylated, and that lack of this regulation in *Ate1* knockout cells directly inhibits the activity of these components. This possibility constitutes an exciting direction for future studies.

To date, very few ATE1-interacting proteins have been identified. Our finding that a fraction of ATE1 can bind to RARS adds an important interaction partner to this very short list. In principle, RARS binding can ensure that ATE1 is strategically placed to utilize newly conjugated arginyl-tRNA^Arg^ before it can be used for translation. Even though our study suggests that RARS availability is not rate-limiting for arginylation, this mechanism can potentially contribute to the regulation of arginylation in vivo.

Given the extremely minor levels of ATE1 found in RARS pulldowns, it is possible that additional unknown players or interactors can participate in the interplay between arginylation and translation. A hypothesis-free discovery approach, such as interaction proteomics, may provide novel candidates for future studies.

Our data suggest that disruption of the MSC can affect ATE1′s redistribution into the cytosol. Previously, two of the four ATE1 isoforms in mammalian cells have been found to exhibit transient localization in the nucleus [11,30,31]. A small fraction of ATE1 has also been found to localize to the mitochondria [34]. Since the displacement of RARS from the MSC in dLZ cells does not affect total ATE1 levels, it is likely that ATE1′s cytosolic increase in these cells occurs due to its redistribution from either the nuclear and/or the mitochondrial fraction into the cytosol. Given that RARS scaffolds the components of the MSC into the nucleus, it is attractive to suggest that it also participates in ATE1′s nuclear shuttling, so that disruption of the RARS nuclear localization would also affect ATE1. This idea is in line with the fact that a fraction of ATE1 can co-immunoprecipitate with RARS. Going further in this reasoning, it is possible that ATE1 may be directly or indirectly linked to the alternative function of RARS in RNA editing and potentially other processes in the nucleus. This possibility of the interplay of ATE1 and RARS’s non-canonical functions constitutes an exciting direction for future studies.

## 4. Materials and Methods

### 4.1. Materials

The human embryonic kidney 293T (HEK293T) parental cells and cells with deletion of the Leu zipper domain in the long RARS (dLZ) [17] were a generous gift from Dr. Paul Schimmel (The Scripps Research Institute, San Diego, CA, USA). Immortalized wild-type and *Ate1* knockout mouse embryonic fibroblasts (MEF) cells were obtained in the lab as described in [8]. Cells were grown in Dulbecco’s modified Eagle’s medium with GlutaMAX supplementation (DMEM + GlutaMAX, Gibco, Waltham, MA, USA) with 10% fetal bovine serum and 1% penicillin–streptomycin at 37°C with 5% CO_2_.

The arginylation sensor plasmids were a generous gift from Dr. Fangliang Zhang (University of Miami, Coral Gables, FL, USA). Plasmids expressing the long and short RARS were generated from a commercial RARS plasmid (HG19710-NY, Sino Biological, Houston, TX, USA) by site-directed mutagenesis in our laboratory.

The following antibodies were used in this study: rabbit anti-R-actin (ABT264 EMD Millipore, 1:2000, Burlington, MA, USA), mouse anti-GFP (Ab1218 Abcam, 1:3000, Cambridge, UK), rabbit anti-RARS (orb247357 Biorbyt, Cambridge, UK, https://www.biorbyt.com/rars-antibody-orb247357.html, accessed on 28 August 2022, 1:5000), mouse anti-puromycin (MABE343 Millipore Sigma, 1:3000), rat anti-ATE1 (homemade, 1:1000), mouse anti-GAPDH (Ab8245 Abcam, 1:5000), mouse anti-alpha tubulin (mAb #3873, Cell Signaling, Danvers, MA, USA) and rabbit anti-histone H3 (#05-928, Sigma, St. Louis, MO, USA).

All the chemicals used for cell treatment, including cycloheximide and chloramphenicol, were obtained from Sigma-Aldrich. Puromycin was purchased from Takara. 

### 4.2. Total Protein Analysis and Cell Fractionation

For total protein analysis, cells were washed once with PBS and harvested by scraping and centrifugation. Cell pellets were lysed in a lysis buffer (50 mM Tris-HCl (pH 7.5), 150 mM NaCl and 0.1% Triton X-100, with a protease inhibitor cocktail (Sigma-Aldrich)) at a 1:5 *w/v* ratio of cell pellet:lysis buffer, followed by vortexing for 5–10 min and sonication (GE Healthcare, Chicago, IL, USA) on ice at Level 5, 20 times (3 s pulse, 3 s pause). Cell lysates were clarified by centrifugation at 16,000× *g* for 15 min at 4 °C. Protein concentrations were measured by the PierceBCA Protein Assay Kit.

Cell fractionation into the nuclear and cytosolic fractions was performed as described in [17] with some modifications. For this, 5 × 10^6^ HEK293T cells were seeded on a 10 cm dish 1 day before the procedure. The cells were washed once with PBS and harvested by scraping and centrifugation. The cell pellet was incubated at −80 °C for 45 min and dissolved into 250 µL of a cell fractionation buffer (20 mM HEPES (pH 7.5), 10 mM KCl, 1 mM MgCl_2_, 1 mM EDTA, 1 mM DDT and complete protease inhibitors). The cells were lysed by passing them through a 27 g needle 20 times and vortexing for 30 s. The sample was centrifuged at 800× *g* for 5 min at 4 °C. The supernatant was taken carefully and centrifuged further at 16,000× *g* for 15 min at 4 °C. The supernatant was mixed with 4× SDS loading buffer, followed by boiling for 10 min, then 10 µL of cytoplasmic fraction was loaded for SDS-PAGE electrophoresis.

### 4.3. Cell Treatments for Arginylation Sensor Measurements, Translation Inhibition and the Puromycylation Assay

Cell transfections were performed in 6-well plates using Lipofectamine 2000 (Thermo Fisher Scientific, Waltham, MA, USA) according to the manufacturer’s protocol, using a 1:3 ratio of DNA:Lipofectamine (4 µg DNA: 12 µL Lipofectamine).

For the arginylation sensor experiments, cells were transfected with the arginylation sensor plasmid 24–48 h prior to the experiment. For arginylation measurements in the confluent and semiconfluent cultures shown in Figure 1, cells transfected with the sensor were split after 24 h to achieve 50% and 100% confluency and incubated for a further 24 h in the culture. In Figure 2A, 48 h after transfection, 100 µg/mL cycloheximide and 40 µg/mL chloramphenicol were added to the culture media, followed by further incubation for 1 h. In Figure 3C, an equimolar mixture of arginylation sensor and RARS plasmids was transfected into the cells 48 h prior to harvesting.

On the day of the experiment, the transfected cells were washed once with phosphate-buffered saline (PBS, Corning, Corning, NY, USA) and harvested by scraping and centrifugation. Cell pellets were lysed in a 4 × SDS loading buffer at a 1:20 *w/v* ratio (1 mg cell pellet: 20 µL buffer), followed by boiling the samples for 10 min. Next, 10 µL of each sample was loaded for SDS-PAGE electrophoresis and analyzed by Western blotting.

For the puromycylation assay, wild-type and *Ate1*^−/−^ MEFs were treated with 10 µg/mL puromycin for 15 min at 37 °C. Cells were collected and lysed by sonication to measure the total protein concentration using the Pierce BCA Protein Assay Kit. Here, 16 µg total protein/lane of each sample was loaded for SDS-PAGE electrophoresis.

### 4.4. Immunoprecipitation

For anti-RARS immunoprecipitation (IP), dLZ or parental HEK293T cells were grown in 10 cm dishes to 60–80% confluency and transfected with plasmids expressing the short, long or wild-type RARS as indicated in the figures. 48 h aftertransfection, the cells were harvested into a lysis buffer and sonicated as described above in the “Total Protein Analysis” section. 50 µL of Protein A agarose beads (Invitrogen, Waltham, MA, USA) pre-equilibrated with the lysis buffer were added to clear up the lysate for 1 h at room temperature on a rocker. Following the removal of the beads by centrifugation, 10 µg of anti-RARS antibody (orb247357 Biorbyt) was added to the cell lysate and incubated on a rocker for 1 h at room temperature, followed by the addition of 50 µL of Protein A agarose beads (Invitrogen) pre-equilibrated in the lysis buffer and additional incubation for 1 h at room temperature on a rocker. The beads were collected by centrifugation and washed three times with the lysis buffer. Next, 20 µL of a 4× SDS loading buffer was added to the beads and boiled, followed by analysis by SDS-PAGE electrophoresis and Western blotting.

For the ATE1–RARS binding assays shown in Figure 4, 5 μg of purified ATE1 and 5 μg of purified short RARS prepared by *E. coli* expression of His-tagged recombinant proteins as described in [35] were mixed in a 120 μL reaction in a lysis buffer with or without the addition of 2 μg of tRNA (overexpressed and purified from *E. coli* as described in [36]), 100 μg of RNase A (ThermoFisher) and/or the cell extract (prepared as described above in the “Total Protein Analysis” section from wild-type MEFs). Next, 50 µL of Protein A agarose beads (Invitrogen) pre-equilibrated with the lysis buffer was added to clear the reaction for 2 h at 4 °C on a rocker. Following removal of the beads by centrifugation, 3 µg of anti-RARS antibody (orb247357 Biorbyt) was added to the supernatant and incubated on a rocker for 1 h at 4 °C, followed by the addition of 50 µL of Protein A agarose beads (Invitrogen) pre-equilibrated in the lysis buffer and additional incubation overnight at 4 °C on a rocker. The beads were collected by centrifugation and washed three times with the lysis buffer. Next, 50 µL of a 4× SDS loading buffer was added to the beads and boiled, followed by analysis by SDS-PAGE electrophoresis and Western blotting.

### 4.5. Western Blotting

The gels were transferred to a nitrocellulose membrane at 100 V for 60 min and stained with Ponceau S to detect total protein levels. For protein level quantification using Ponceau S staining, the images were converted to black and white, inverted so that the protein bands were seen as white on a black background and quantified as total gray level in the entire lane the using “integrated density” function in Adobe Photoshop. The blots were blocked with 5% milk in PBS-Tween at 4 °C for 16 h and incubated with primary antibodies for 60 min at room temperature, followed by washing and incubation with secondary antibodies (1:5000) conjugated to IRDye800 or IRDye680. Images were acquired and analyzed by the Odyssey Imaging System (LI-COR, Lincoln, NE, USA).

## Data Availability

Not applicable.

## References

[1] Saha S., Kashina A. (2011). Posttranslational arginylation as a global biological regulator. Dev. Biol..

[2] Wang J., Han X., Leu N.A., Sterling S., Kurosaka S., Fina M., Lee V.M., Dong D.W., Yates J.R., Kashina A. (2017). Protein arginylation targets alpha synuclein, facilitates normal brain health, and prevents neurodegeneration. Sci. Rep..

[3] Cornachione A.S., Leite F.S., Wang J., Leu N.A., Kalganov A., Volgin D., Han X., Xu T., Cheng Y.S., Yates J.R. (2014). Arginylation of myosin heavy chain regulates skeletal muscle strength. Cell Rep..

[4] Wang J., Han X., Wong C.C., Cheng H., Aslanian A., Xu T., Leavis P., Roder H., Hedstrom L., Yates J.R. (2014). Arginyltransferase ATE1 catalyzes midchain arginylation of proteins at side chain carboxylates in vivo. Chem Biol..

[5] Zhang F., Saha S., Kashina A. (2012). Arginylation-dependent regulation of a proteolytic product of talin is essential for cell-cell adhesion. J. Cell Biol..

[6] Saha S., Wong C.C., Xu T., Namgoong S., Zebroski H., Yates J.R., Kashina A. (2011). Arginylation and methylation double up to regulate nuclear proteins and nuclear architecture in vivo. Chem. Biol..

[7] Wong C.C.L., Xu T., Rai R., OBailey A., Yates J.R., Wolf Y., Zebroski H., Kashina A. (2007). Global analysis of posttranslational protein arginylation. PLoS Biol..

[8] Karakozova M., Kozak M., Wong C.C., Bailey A.O., Yates J.R., Mogilner A., Zebroski H., Kashina A. (2006). Arginylation of beta-actin regulates actin cytoskeleton and cell motility. Science.

[9] Zhang F., Patel D.M., Colavita K., Rodionova I., Buckley B., Scott D.A., Kumar A., Shabalina S.A., Saha S., Chernov M. (2015). Arginylation regulates purine nucleotide biosynthesis by enhancing the activity of phosphoribosyl pyrophosphate synthase. Nat. Commun..

[10] Rai R., Zhang F., Colavita K., Leu N.A., Kurosaka S., Kumar A., Birnbaum M.D., Győrffy B., Dong D.W., Shtutman M. (2016). Arginyltransferase suppresses cell tumorigenic potential and inversely correlates with metastases in human cancers. Oncogene.

[11] Rai R., Kashina A. (2005). Identification of mammalian arginyltransferases that modify a specific subset of protein substrates. Proc. Natl. Acad. Sci. USA.

[12] Avcilar-Kucukgoze I., Gamper H., Polte C., Ignatova Z., Kraetzner R., Shtutman M., Hou Y.M., Dong D.W., Kashina A. (2020). tRNA(Arg)-Derived Fragments Can Serve as Arginine Donors for Protein Arginylation. Cell Chem. Biol..

[13] Zheng Y.G., Wei H., Ling C., Xu M.G., Wang E.D. (2006). Two forms of human cytoplasmic arginyl-tRNA synthetase produced from two translation initiations by a single mRNA. Biochemistry.

[14] Sivaram P., Deutscher M.P. (1990). Existence of two forms of rat liver arginyl-tRNA synthetase suggests channeling of aminoacyl-tRNA for protein synthesis. Proc. Natl. Acad. Sci. USA.

[15] Mirande M. (2017). The Aminoacyl-tRNA Synthetase Complex. Subcell Biochem..

[16] Fu Y., Kim Y., Jin K.S., Kim H.S., Kim J.H., Wang D., Park M., Jo C.H., Kwon N.H., Kim D. (2014). Structure of the ArgRS-GlnRS-AIMP1 complex and its implications for mammalian translation. Proc. Natl. Acad. Sci. USA.

[17] Cui H., Kapur M., Diedrich J.K., Yates J.R., Ackerman S.L., Schimmel P. (2021). Regulation of ex-translational activities is the primary function of the multi-tRNA synthetase complex. Nucleic Acids Res..

[18] Nathanson L., Deutscher M.P. (2000). Active aminoacyl-tRNA synthetases are present in nuclei as a high molecular weight multienzyme complex. J. Biol. Chem..

[19] Bachmair A., Finley D., Varshavsky A. (1986). In vivo half-life of a protein is a function of its amino-terminal residue. Science.

[20] Varshavsky A. (2000). Ubiquitin fusion technique and its descendants. Methods Enzymol..

[21] Zhang F., Saha S., Shabalina S.A., Kashina A. (2010). Differential arginylation of actin isoforms is regulated by coding sequence-dependent degradation. Science.

[22] Kumar A., Birnbaum M.D., Patel D.M., Morgan W.M., Singh J., Barrientos A., Zhang F. (2016). Posttranslational arginylation enzyme Ate1 affects DNA mutagenesis by regulating stress response. Cell Death Dis..

[23] Chen L., Kashina A. (2019). Quantification of intracellular N-terminal beta-actin arginylation. Sci. Rep..

[24] Bereiter-Hahn J., Munnich A., Woiteneck P. (1998). Dependence of energy metabolism on the density of cells in culture. Cell Struct. Funct..

[25] Aviner R. (2020). The science of puromycin: From studies of ribosome function to applications in biotechnology. Comput. Struct Biotechnol. J..

[26] Nathans D. (1964). Puromycin Inhibition of Protein Synthesis: Incorporation of Puromycin into Peptide Chains. Proc. Natl. Acad. Sci. USA.

[27] Yarmolinsky M.B., Haba G.L. (1959). Inhibition by Puromycin of Amino Acid Incorporation into Protein. Proc. Natl. Acad. Sci. USA.

[28] Schmidt E.K., Clavarino G., Ceppi M., Pierre P. (2009). SUnSET, a nonradioactive method to monitor protein synthesis. Nat. Methods..

[29] Ciechanover A., Ferber S., Ganoth D., Elias S., Hershko A., Arfin S. (1988). Purification and characterization of arginyl-tRNA-protein transferase from rabbit reticulocytes. Its involvement in post-translational modification and degradation of acidic NH2 termini substrates of the ubiquitin pathway. J. Biol. Chem..

[30] Wang J., Han X., Saha S., Xu T., Rai R., Zhang F., Wolf Y.I., Wolfson A., Yates J.R., Kashina A. (2011). Arginyltransferase is an ATP-independent self-regulating enzyme that forms distinct functional complexes in vivo. Chem. Biol..

[31] Kwon Y.T., Kashina A.S., Varshavsky A. (1999). Alternative splicing results in differential expression, activity, and localization of the two forms of arginyl-tRNA-protein transferase, a component of the N-end rule pathway. Mol. Cell Biol..

[32] Fissolo S., Bongiovanni G., Decca M.B., Hallak M.E. (2000). Post-translational arginylation of proteins in cultured cells. Neurochem. Res..

[33] Evans M., Clark W.C., Zheng G., Pan T. (2017). Determination of tRNA aminoacylation levels by high-throughput sequencing. Nucleic Acids Res..

[34] Jiang C., Moorthy B.T., Patel D.M., Kumar A., Morgan W.M., Alfonso B., Huang J., Lampidis T.J., Isom D.G., Barrientos A. (2020). Regulation of Mitochondrial Respiratory Chain Complex Levels, Organization, and Function by Arginyltransferase 1. Front. Cell Dev. Biol..

[35] Wang J., Kashina A.S. (2015). Bacterial Expression and Purification of Recombinant Arginyltransferase (ATE1) and Arg-tRNA Synthetase (RRS) for Arginylation Assays. Methods Mol. Biol..

[36] Avcilar-Kucukgoze I., Gamper H., Hou Y.-M., Kashina A. (2020). Purification and Use of tRNA for Enzymatic Post-translational Addition of Amino Acids to Proteins. STAR Protoc..

